# In vitro gametogenesis: Towards competent oocytes: Limitations and future improvements for generating oocytes from pluripotent stem cells in culture

**DOI:** 10.1002/bies.202400106

**Published:** 2024-11-05

**Authors:** Eishi Aizawa, Antoine H. F. M. Peters, Anton Wutz

**Affiliations:** ^1^ Institute of Molecular Health Sciences Swiss Federal Institute of Technology, ETH Zurich Zurich Switzerland; ^2^ RIKEN Center for Biosystems Dynamics Research Kobe Japan; ^3^ Friedrich Miescher Institute for Biomedical Research Basel Switzerland; ^4^ Faculty of Sciences University of Basel Basel Switzerland

**Keywords:** cell culture, gametogenesis, germline, mouse development, oocyte, stem cells

## Abstract

Production of oocytes from pluripotent cell cultures in a dish represents a new paradigm in stem cell and developmental biology and has implications for how we think about life. The spark of life for the next generation occurs at fertilization when sperm and oocyte fuse. In animals, gametes are the only cells that transmit their genomes to the next generation. Oocytes contain in addition a large cytoplasm with factors that direct embryonic development. Reconstitution of mouse oocyte and embryonic development in culture provides experimental opportunities and facilitates an unprecedented understanding of molecular mechanisms. However, the application of in vitro gametogenesis to reproductive medicine or infertility treatment remains challenging. One significant concern is the quality of in vitro‐derived oocytes. Here, we review the current understanding and identify limitations in generating oocytes in vitro. From this basis, we explore opportunities for future improvements of the in vitro approach to generating high‐quality oocytes.

## Graphical abstract

Functional gametes can be obtained by differentiation of pluripotent stem cells in culture. Here we review challenges faced by current approaches for obtaining developmentally competent oocytes from mouse embryonic and induced pluripotent stem cells. We consider details of germline development to suggest future improvements of in vitro oogenesis.

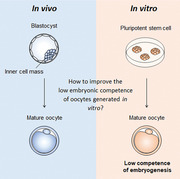

## INTRODUCTION

Recent progress in stem cell biology has led to an impressive advance in generating tissues and fully functional cell types in culture. These approaches are based on pluripotent stem cells (PSCs) and extend the entire span of development from unspecified epiblast to recapitulate cell lineage development and morphology of the embryo. Notable examples are the recent reconstitution of post‐implantation embryos,^[^ ^1^, ^2^ ^]^ the reconstitution of renal tissues resembling glomeruli from three populations of progenitors obtained from PSCs,^[^ ^3^ ^]^ and the production of spermatids and oocytes entirely in culture from PSCs that are potent to support development of healthy mice after fertilization.^[^ ^4^, ^5^, ^6^ ^]^ We focus on the last as oocytes from PSCs have been obtained that are equivalent to oocytes from the living organism.^[^ ^7^ ^]^ The proof of equivalence of in vitro‐derived and embryonic cells has been difficult in some systems. For example rigorous testing of hematopoietic stem cells (HSCs) by engraftment indicates that deficits still exist and PSC‐derived HSCs do not perform as well as definitive HSCs from the embryonic aorta.^[^ ^8^ ^]^ The generation of oocytes from PSCs that support development of healthy mice leaves no doubt of equivalence and ushers in a new area for developmental biology. Reconstitution of development from early epiblast cells facilitates testing of our understanding of mechanisms of development of tissues, organs, and entire embryos. Developmental and stem cell biology offer an extensive knowledge base for reconstitution efforts, which have begun to inspire new technological applications. In the following we focus on mouse oocyte development as a paradigm for reconstitution experiments with the aim to identify needs and opportunities for further improvement and provide a perspective on future directions.

## OVERVIEW OF FEMALE GERM CELL DEVELOPMENT IN MICE

**FIGURE 1:**
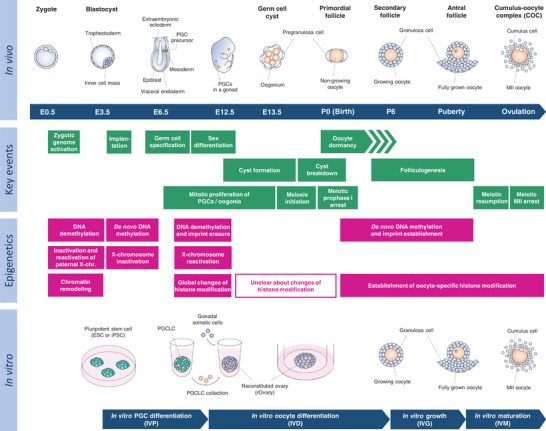
**Overview of female mouse germ cell development in vivo and in vitro**. (top) A schematic illustration of female germ cell development in mice. After embryo implantation, PGC precursors arise in the proximal epiblast and migrate to gonads. Upon sex determination female PGCs develop as oogonia, and germ cell cysts form with oogonia clustering through proliferation. Subsequent breakdown of these cysts results in the formation of primordial follicles housing non‐growing oocytes. A subset of primordial follicles undergoes folliculogenesis, progressing to antral follicles. Eventually, the cumulus‐oocyte complex (COC) is released from an antral follicle, leading to the emergence of mature MII oocytes. (middle) The middle part indicates key biological events, related to embryonic development, germ cell characterization, meiosis, and oocyte development, along with the timeline of in vivo development (green). Also, corresponding epigenetic events including DNA methylation, X‐chromosome inactivation and histone modification are indicated in magenta color. (bottom) A schematic illustration of germ cell development from pluripotent stem cells (PSCs) in the culture system established by Hikabe et al.^[^ ^4^ ^]^ PSCs differentiate to PGCLCs by in vitro PGC differentiation (IVP). PGCLCs sorted from cell aggregates are co‐cultured with somatic cells collected from fetal gonads, leading to the formation of a reconstituted ovary (rOvary). Primary or secondary follicles appear in the rOvary during in vitro oocyte differentiation (IVD). Isolation and subsequent culture of follicles lead to the development of fully grown oocytes and MII oocytes by in vitro growth (IVG) and in vitro maturation (IVM), respectively. E, embryonic day; ESC, embryonic stem cell; iPSC, induced pluripotent stem cell; P, postnatal day; PGC, primordial germ cell; PGCLC, PGC‐like cell; MII, metaphase II.

The mouse germline segregates from the proximal posterior post‐implantation epiblast^[^ ^9^, ^10^ ^]^ (Figure 1). Its establishment can be conceptually described as a series of separate key steps.^[^ ^11^ ^]^ Germ cell fate is induced in proximal epiblast cells in response to bone morphogenetic protein (BMP) signaling at embryonic day (E) 6.0‐6.5.^[^ ^12^ ^]^ BMP4 from the extraembryonic ectoderm transiently induces a mesodermal pathway and expression of *Brachyury* (*T*), followed by expression of *Blimp1* (*Prdm1*) and *Prdm14*.^[^ ^13^ ^]^ It has been shown that *Blimp1* expression correlates with a reduced phosphorylation of Smad1, Smad5, or Smad9 in early PGCs within the extraembryonic mesoderm in the posterior proximal region of the E6.5 embryo.^[^ ^14^ ^]^ PGCs appear to be unresponsive to the high levels of different signaling molecules in the mesodermal niche and avoid entering somatic differentiation. PGC determination is marked by expression of *AP2γ* (*Tfap2c*) and *Stella* (*Dppa3*) in the extraembryonic mesoderm at the base of the allantois around E7.25, when a transcriptional network of pluripotent cells is re‐established with expression of *Sox2*, *Nanog*, and *Oct4*. Subsequently, PGCs migrate through the basement membrane and integrate into the endoderm of the forming hindgut. During migration dramatic genomic changes occur including reactivation of the inactive X chromosome, loss of DNA methylation, and changes in post‐translational histone modifications^[^ ^15^ ^]^ From E9.5 PGCs reach the genital ridges, which form from the coelomic lining of intermediate mesoderm at a mesonephric position. It is important to note that neither sex determination nor entry into meiosis has occurred at this stage. To account for this, the conceptual stage of gametogenesis competent cells (GCCs) for PGCs within the developing gonad at E10.5‐11.5 has been considered.^[^ ^16^ ^]^ The expression of *Dazl* plays a key role for the transition from PGC to GCC, which are capable of initiating either oogenic or spermatogenic fate in response to appropriate gonadal cues.

Sex determination around E11.5 splits the developmental trajectories and leads to morphologically distinct male and female gonads by E12.5. PGCs initiate sexually dimorphic development by signals from somatic cells in the gonad.^[^ ^17^, ^18^ ^]^ The oogenic fate of PGCs is determined by expression of the transcriptional regulator ZGLP1.^[^ ^19^ ^]^ Bone morphogenetic proteins (BMPs), expressed in pregranulosa cells in the gonad, induce *Zglp1* expression in PGCs.^[^ ^20^ ^]^ PGCs form oogonia and continue mitotic proliferation before entering meiosis around E13.5. Incomplete cytokinesis in proliferating oogonia leads to the formation of clusters of germ cells with intercellular bridges between daughter cells, which are called germ cell cysts.^[^ ^21^ ^]^ BMP signaling confers meiotic competence to oogonia, while the initiation of meiosis is activated by retinoic acid (RA) from the gonadal environment, which induces *Stra8* expression in oogonia.^[^ ^22^ ^]^ Oogonia proceed through stages of leptotene, zygotene and pachytene, and arrest at the diplotene in meiotic prophase I. Simultaneously, oogonia develop to primary oocytes surrounded by pregranulosa cells, leading to the formation of primordial follicles around birth. Notably, meiosis can be separated from oocyte development. In *Stra8* mutant mice morphologically well‐developed oocytes have been observed without entry into meiosis.^[^ ^23^ ^]^ Furthermore, expression of a set of transcription factors in PSCs has been shown to allow differentiation into morphologically well‐developed oocytes without meiotic entry.^[^ ^24^ ^]^ Therefore, meiosis and oocyte development can be seen as separate molecular processes.

After birth, most primordial follicles pause their development at the dormant state in meiotic prophase I, while a subset resumes folliculogenesis in response to hormones and intra‐follicular signaling^[^ ^25^ ^]^ (Figure 1). It is generally believed that primordial follicles are not replenished after birth, therefore females are born with a finite pool of oocytes. Once awakened from the dormant state, flattened pregranulosa cells in primordial follicles differentiate to cuboidal granulosa cells and undergo proliferation to form primary and then secondary follicles. Bidirectional communication between oocytes and surrounding somatic cells facilitates further development towards antral follicles.^[^ ^26^ ^]^ GDF9 and BMP15, expressed by oocytes, play a central role by guiding the production of protein signals from granulosa cells and the differentiation of theca cells.^[^ ^27^, ^28^ ^]^ Under the regulation of the hypothalamic‐pituitary‐ovarian axis, female hormones such as follicle‐stimulating hormone (FSH) and luteinizing hormone (LH) prompt the selection of dominant antral follicles for ovulation.^[^ ^29^ ^]^ This process initiates the maturation of the oocyte, including the resumption of meiosis in fully grown oocytes towards the metaphase II (MII), culminating in the release of cumulus‐oocyte complexes (COC). DNA methylation is de novo established on the oocyte genome in growing oocytes after primordial follicles exited the dormant state.^[^ ^30^ ^]^ DNA methylation at imprinting control regions established in growing oocytes plays a pivotal role in the parent‐specific expression of imprinted genes post‐fertilization.^[^ ^31^ ^]^ Moreover, during their growth, oocytes acquire a distinct chromatin configuration. Acquisition of H3K27me3 in broad genomic regions of oocytes contributes to regulating paternal X‐chromosome inactivation and maternal allele‐specific repression of dozens of genes, as non‐canonical imprinting, which for some genes is essential for proper placental development.^[^ ^32^, ^33^ ^]^

**FIGURE 2:**
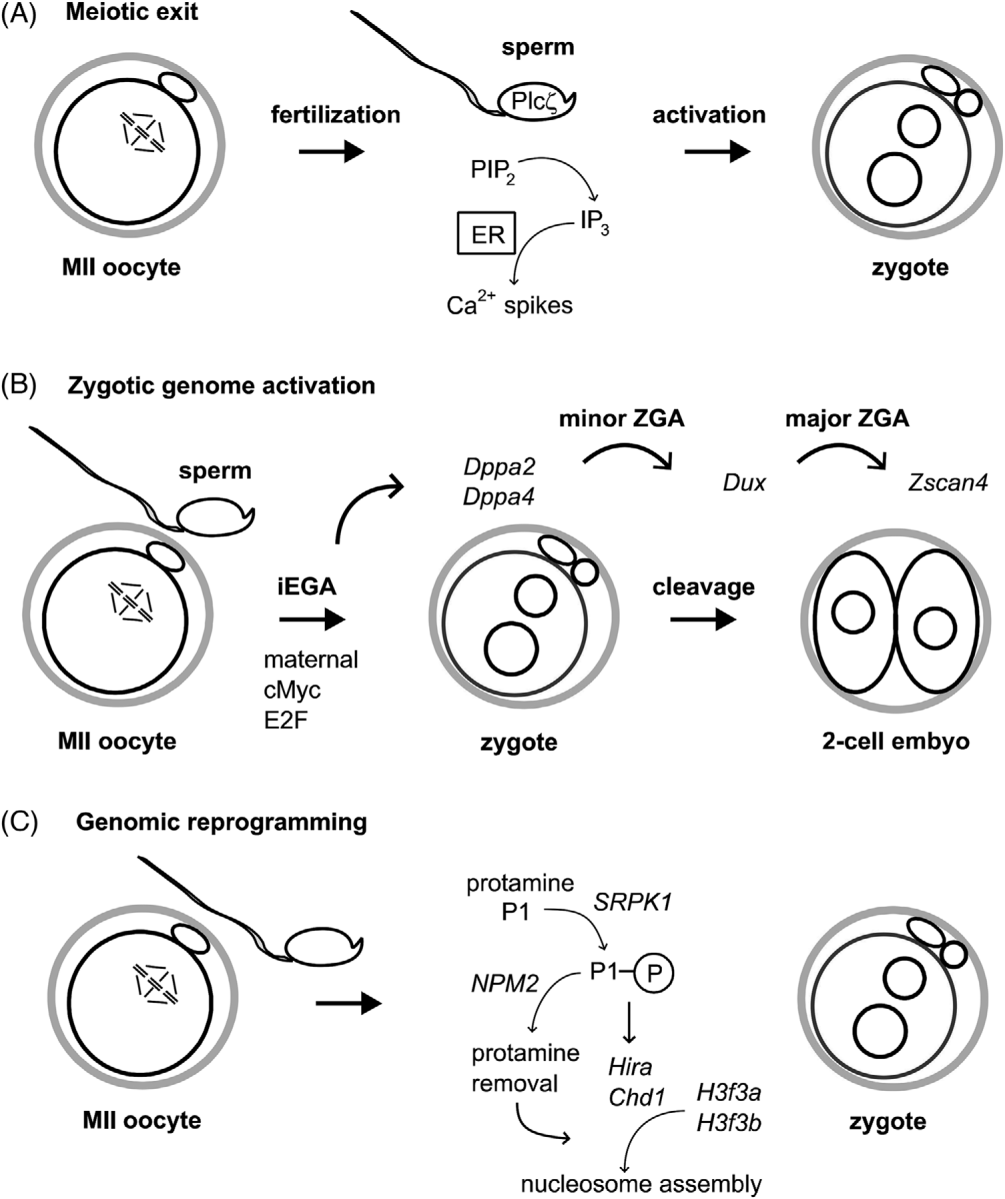
**The mature MII oocyte supports a number of critical processes after fertilization**. (A) Phospholipase C zeta (PLCζ) released from sperm cleaves phosphatidylinositol biphosphate (PIP_2_) into inositol triphosphate (IP_3_) triggering the release of calcium ions (Ca^2+^) from the endoplasmic reticulum (ER) that lead to re‐entry into the cell cycle.^[^ ^36^ ^]^ Progression of the second meiotic division separates sister chromatids of the maternal chromosomes into the maternal pronucleus and the second polar body by asymmetric cell division. (B) Transcription is initiated throughout the zygotic genome, referred to as zygotic genome activation (ZGA)^[^ ^119^, ^120^ ^]^. After initial embryonic genome activation (iEGA),^[^ ^121^ ^]^ and a minor wave of ZGA, the transcription factor *Dux* subsequently contributes to further gene activation at the major wave of ZGA at the 2‐cell stage.^[^ ^122^, ^123^ ^]^ *Zscan4* is a ZGA specific gene that is specifically expressed at the 2‐cell stage.^[^ ^119^ ^]^ (C) The sperm DNA is packaged on protamines, which are replaced by histones. Serine/arginine‐rich splicing factor protein kinase 1 (*Srpk1*) phosphorylates protamine P1 facilitating its removal by *Nucleoplasmin* 2 (*Npm2*). Phosphorylated P1 also recruits HIRA that is a component of a chromosome assembly complex.^[^ ^124^ ^]^ HIRA incorporates histone H3.3, which is encoded by the *H3f3a* and *H3f3b* genes, initiating nucleosomal packaging of the paternal chromosomes in the paternal pronucleus.

The mature oocyte is a highly specialized cell that is essential for establishing a totipotent zygote and initiation of embryonic development. Maternal factors including RNAs and proteins in the cytoplasm and subcortical maternal complex support a number of molecular mechanisms that are triggered by fertilization.^[^ ^34^, ^35^ ^]^ At fertilization, phospholipase C zeta (PLCζ) from the sperm head triggers Ca^2+^ oscillation in the zygote, which resumes meiosis leading to polar body extrusion^[^ ^36^ ^]^ (Figure 2A). A substantial amount of maternal mRNA is present in the zygote, which is subsequently degraded by the end of the 2‐cell stage and supplanted by zygotic transcripts in a process known as the maternal‐to‐zygotic transition (MZT).^[^ ^37^ ^]^ A minor wave of zygotic genome activation (ZGA) occurs during the S‐phase of the zygote^[^ ^38^, ^39^ ^]^ and a major wave of ZGAs during the mid‐to‐late 2‐cell stage (Figure 2B). Fertilization also triggers genomic reprogramming which involves the repackaging of the sperm genome from protamines into nucleosomes and histone exchange on the maternal chromosomes (Figure 2C). Genomic reprogramming also involves metabolic regulation and continues over subsequent preimplantation development. In particular DNA methylation is removed from the embryonic genome. Several parallel processes contribute to initiating the embryonic development once the oocyte has been released from cell cycle arrest.^[^ ^40^ ^]^

## ADVANCES OF IN VITRO CULTURE FOR MOUSE OOCYTE DEVELOPMENT

The advancement of in vitro culture systems for oocyte development has been a significant journey, evolving for nearly a century to the cutting‐edge methodologies of today (Table 1). The primary objective has been to understand the mechanism of germ cell development, offering insights into developmental and reproductive biology. Nowadays, future applications in medicine, infertility treatment, and animal conservation efforts are also considered.^[^ ^7^ ^]^

**TABLE 1:** Summary of key studies on reconstitution of mouse germ cell development.

Reference	Origin	Sex of orign	Cell type obtained	Strategy for development	Functional assay of cells obtained
Martinovitch, 1938 [1]	Rats and mice ovaries from E16 to P4	Female	Oocyte	Culture of whole ovaries on watchglass chambers for up to 30 days	Not tested
Blandau et al., 1965 [2]	E16 fetal ovary	Female	Oocyte	Culture of E16 fetal ovarian fragments on rose chambers for more than 50 days	Not tested
Eppig and O'Brien, 1996 [3]	P6, 8 and 12 ovaries	Female	Mature oocyte	Two‐step cultures for organ and follicle development for a total of 22 days	Blastocyst development
Obata et al., 2002 [4]	E12.5 gonad	Female	Reconstituted functional oocyte	Gonad culture; tansfer of in vitro‐derived oocyte nucleus to enucleated fully grown oocyte	Offspring delivery
Hübner et al., 2003 [5]	ESC	Male	Oocyte‐like cell	Spontaneous differentiation without growth factors in adherent cultures	Parthenogenic blastocyst‐like formation
O'Brien et al., 2003 [6]	P0 ovary	Female	Mature oocyte	Revision of a two‐step culture reported previously (Eppig and O'Brien, 1996)	Offspring delivery
Toyooka et al., 2003 [7]	ESC	Male	MVH‐positive cell; sperm after transplantation	EB differentiation and co‐culture with cells producing BMP4	Sperm development
Geijsen et al., 2004 [8]	ESC	Male	Spermatid‐like haploid cell	EB differentiation and RA treatment	Blastocyst development
Lacham‐Kaplan et al., 2006 [9]	ESC	Male	Oocyte‐like cell	EB culture with testicular cell conditioned medium	Not tested
Nayernia et al., 2006 [10]	ESC	Male	*Stra8*‐positive cell; sperm after transplantation	Culture on a feeder layer with RA treatment; Transplantation into seminiferous tubles	Offspring delivery (premature death)
Qing et al., 2007 [11]	ESC	Male	MVH‐ and GDF9‐positive oocyte‐like cell	EB formation and co‐culture with ovarian granulosa cells	Not tested
Eguizabal et al., 2009 [12]	ESC, EGC	Female, male	POU5F1‐ and MVH‐positive cell; SCP3‐positive cell	EB formation and suspension culture, followed by RA treatment or culture with CHO cells	Not tested
Hayashi et al., 2009 [13]	EpiSC from E6.5 Epiblast	Not specified	*Blimp1*‐ or *Stella*‐positive cells (PGCLC); oocytes after co‐culture	Culture in medium containing BMP4, followed by co‐culture with E12.5 female gonadal cells	EGC derivation; oocyte development
Ohinata et al., 2009 [14]	E5.25 ‐ 6.75 epiblast	Male	*Blimp1*‐ and *Stella*‐positive cells (PGCLC); spermatozoa after transplantation	Culture in medium containing BMP4/8b; transplantation into seminiferous tubles	Offspring delivery
Young et al., 2010 [15]	ESC	Male	POU5F1‐positive cells with elevation of germ cell markers	EB formation with BMP2/4/8b	Not tested
Hayashi et al., 2011 [16]	ESC, E5.75 Epiblast, EpiSC	Male	PGCLC; spermatozoa after transplantion	EpiLC differentiation; PGCLC differentiation; transplantation into seminiferous tubules	Offspring delivery
Hayashi et al., 2012 [17]	ESC, iPSC	Female	PGCLC; oocyte after transplantion	EpiLC differentiation; PGCLC differentiation; co‐culture with gonadal somatic cells followed by transplantation into ovary	Offspring delivery
Zhu et al., 2012 [18]	iPSC	Male	Spermatids after transplantation	EB formation and RA treatment, followed by transplantation into seminiferous tubles	Not tested
Nakaki et al., 2013 [19]	ESC	Male	PGCLC; spermatozoa after transplantation	EpiLC differentiation, followed by overexpression of transcriptional factors	Offspring delivery
Kimura et al., 2014 [20]	ESC	Male	PGCLC	Co‐culture with OP9 feeders and MEK inhibitor treatment	EGC derivation
Hikabe et al., 2016 [21]	ESC, iPSC	Female	Mature oocyte	PGCLC differentiation, followed by co‐culture with gonadal somatic cells	Offspring delivery
Ishikura et al., 2016 [22]	ESC	Male	Spermatogonia; spermatozoa after transplantation	PGCLC differentiation, followed by co‐culture with gonadal somatic cells; transplantation of spermatogonia‐like cells	Offspring delivery
Morohaku et al., 2016 [23]	E12.5 gonad	Female	Mature oocyte	Two step‐culture using collagenase and polyvinylpyrrolidone	Offspring delivery
Zhou et al., 2016 [24]	ESC	Male	Spermatid	PGCLC differentiation, followed by co‐culture with neonatal testicular somatic cells	Offspring delivery
Miyauchi et al., 2017 [25]	ESC	Female	Fetal oocyte	PGCLC differentiation, followed by PGCLC propagation and treatment with RA and BMP2	Not tested
Ohta et al., 2017 [26]	ESC	Female, male	Expanded PGCLC	PGCLC differentiation, followed by co‐culture with m220 feeders supplemented with rolipram and forskolin	Not tested
Nagamatsu et al., 2019 [27]	E12.5 gonad	Female	Dormant oocyte	Culture under exogenous pressure	Not tested
Shimamoto et al., 2019 [28]	ESC	Female	Dormant oocyte	PGCLC differentiation; culture under a hypoxia condition during the IVD	Not tested
Nagaoka et al., 2020 [29]	ESC	Female	Fetal oocyte	PGCLC differentiation, followed by PGCLC propagation and overexpression of *Zglp1*	Not tested
Hamazaki et al, 2021 [30]	ESC, iPSC	Female	Oocyte‐like cell	Overexpression of transcription factors in ESC/iPSC	Cleavages after fertilization
Ishikura et al., 2021 [31]	ESC	Male	Spermatozoon	PGCLC differentiation; PGCLC propagation; co‐cultue with gonadal somatic cells; culture within seminiferous tubules	Offspring delivery
Yoshino et al., 2021 [32]	ESC	Female	Mature oocyte	PGCLC and FOSLC differentiation, followed by co‐culture of these 2 cell types	Offspring delivery
Aizawa et al., 2023 [33]	ESC, iPSC	Female	Mature oocyte	PGCLC differentiation, followed by co‐culture with gonadal somatic cells	Blastocyst development
Murakami et al., 2023 [34]	ESC, iPSC	Male	Mature oocyte	Loss of Y chromosome and X chromosome duplication in ESC/iPSC; PGCLC differentiation; co‐culture with gonadal somatic cells	Offspring delivery

CHO, Chinese hamster ovary; EB, embryoid body; EGC, embryonic germ cell; EpiLC, epiblast‐like cell; EpiSC, epiblast stem cell; ESC, embryonic stem cell;

FOSLC, fetal ovarian somatic cell‐like cell; iPSC, induced pluripotent stem cell; IVD, in vitro oocyte development; PGC, primordial germ cell; PGCLC, PGC‐like cell; RA, retinoic acid.

The first noteworthy study was reported in 1938, which observed oocyte development from rat and mouse ovaries harvested between E16 and postnatal day (P) 4.^[^ ^41^ ^]^ The whole ovary was cultured with medium consisting of chicken plasma and embryo extract on a glass chamber in a petri dish. An E16 mouse ovary after 19 days of the culture produced oocytes surrounded by no or a single layer of cells. Culture systems to generate mature oocytes from fetal gonads or neonatal ovaries have been largely developed in the late 1900s and the early 2000s.^[^ ^42^, ^43^, ^44^ ^]^ The turn of the 21st century brought a pivotal shift with the emergence of PSCs, including embryonic stem cells (ESCs), epiblast stem cells (EpiSCs), embryonic germ cells (EGCs), and induced pluripotent stem cells (iPSCs). Several studies have reported the generation of oocytes or spermatid‐like cells by pursuing methods of undirected differentiation, followed by the selection of rare germ cell‐like cells.^[^ ^45^, ^46^, ^47^, ^48^ ^]^ However, these early studies did not obtain healthy offspring from these oocytes or spermatid‐like cells.

**FIGURE 3:**
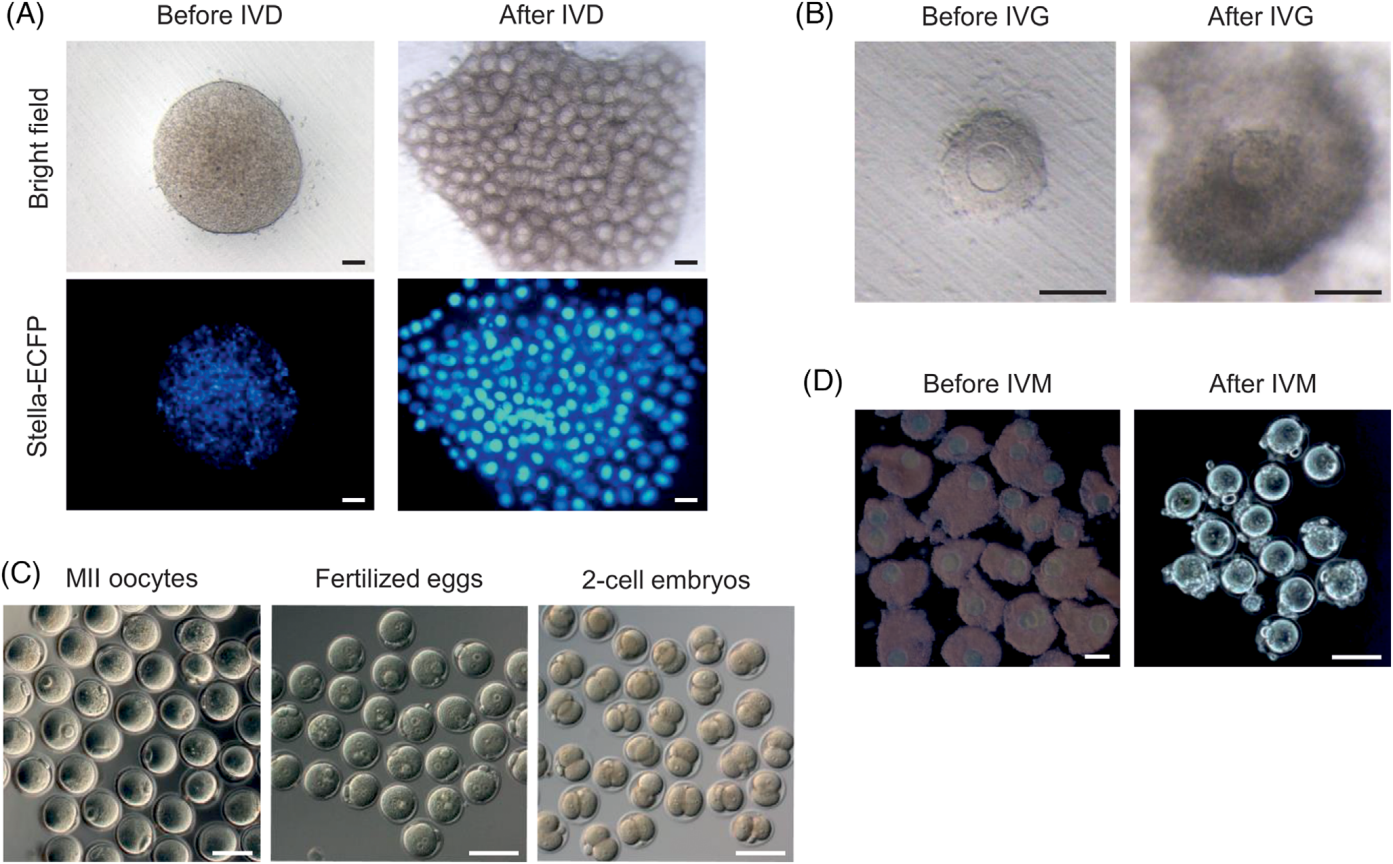
**In vitro oocyte development from ESCs**. All germ cells shown were derived from ESCs bearing *Blimp1*‐Venus (BV) and *Stella*‐ECFP (SC) transgenes. (A) A rOvary development during IVD. Shown are bright field and SC images of a rOvary at day 3 (before IVD) and day 23 (after IVD) after the co‐culture of PGCLCs with gonadal somatic cells. (B) Bright field images of a follicle at day 2 (before IVG) and day 11 (after IVG) of IVG. A round oocyte was observed in the middle of the follicle. Granulosa cells around the oocyte (before IVG) proliferated, forming a cumulus‐oocyte complex (after IVG). (C) Bright field images of MII oocytes after IVM (left), fertilized eggs (middle), and 2‐cell embryos (right) after IVF. (D) COCs and MII oocytes exclusively generated from ESCs. PGCLCs were aggregated with FOSLCs, derived from ESCs bearing *Nr5a1*‐hCD271 and *Foxl2*‐tdTomato, and subsequently developed through IVD and IVG. Shown are a bright field image merged with *Foxl2*‐tdTomato and *Stella*‐ECFP before IVM (left) and a bright field image of MII oocytes after IVM (right). All scale bars, 100 µm. Images of panel A, B, and C are adapted with permission from reference.^[^ ^4^ ^]^ Images of panel D are adapted with permission from reference.^[^ ^5^ ^]^

A breakthrough came with the successful generation of primordial germ cell‐like cells (PGCLCs) from PSCs. Hayashi et al. induced PGCLCs, which were positive for *Blimp1*‐Venus (BV) and *Stella*‐ECFP (SC), using a two‐step culture method with a cocktail of growth factors.^[^ ^49^, ^50^ ^]^ First, PSCs were stimulated with Activin and FGF2 to produce epiblast‐like cells (EpiLCs) bearing properties similar to the epiblast at E5.5 to E6.0. These EpiLCs were then aggregated into embryoid bodies and treated with BMP4 to recapitulate PGC induction. The resulting PGCLCs, isolated using the BV signal and resembling PGCs at the migratory stage around E9.5, developed into spermatozoa and oocytes after transplantation into testes or their aggregates into ovaries. Importantly, these gametes successfully produced healthy offspring, demonstrating their functionality. Since this breakthrough, PGCLC generation has become a common approach for gamete production. Subsequent work by Hikabe et al. demonstrated that the entire female germline development could be recapitulated in vitro (Figure 1).^[^ ^4^ ^]^ Over a 45‐day culture, mature MII oocytes from PSCs could be obtained through four stages: in vitro PGC differentiation (IVP), in vitro oocyte differentiation (IVD), in vitro growth (IVG) and in vitro maturation (IVM). The first stage, IVP, corresponds to PGCLC differentiation from PSCs. PGCLCs were then cultured with somatic cells from E12.5 embryonic gonads to form a reconstituted ovary (rOvary) in low‐binding wells (Figure 3A). During IVD, rOvaries were cultured on a filter membrane, leading to the development of primary or secondary follicles containing growing oocytes. Follicles were subsequently dissected into small groups to stimulate granulosa cell expansion during the IVG (Figure 3B). Finally, expanded follicles underwent IVM, producing mature MII oocytes, which are almost indistinguishable from MII oocytes grown in vivo (Figure 3C). These PSC‐derived MII oocytes were successfully fertilized and developed into full‐term mice after embryo transplantation.

These remarkable advances of in vitro culture systems not only provided functional oocytes but also served as means to investigate various aspects of germ cell development. For instance, the generation of PGCLCs and the establishment of a culture system for PGCLC expansion paved the way for a deeper understanding of sex determination mechanisms in mouse germ cells.^[^ ^51^ ^]^ Studies elucidated the role of BMP signaling and its downstream regulator *Zglp1*, revising a prevailing view to provide an extensive mechanism in determining oogenic fate.^[^ ^19^, ^20^ ^]^ Also, researchers delved into mechanisms of the dormant state of oocytes in primordial follicles, effects of sex chromosomes on oocyte differentiation, and transcription factors involved in oocyte growth.^[^ ^24^, ^52^, ^53^, ^54^ ^]^ Furthermore, a recent innovation has led to the generation of follicles exclusively from PSCs in vitro, eliminating the need for gonadal tissues from fetuses (Figure 3D). Yoshino et al. successfully produced fetal ovarian somatic cell‐like cells (FOSLCs) from mouse ESCs, which supported follicle formation with PGCLCs.^[^ ^5^ ^]^ Notably, functional oocytes have recently been generated from PSCs of male mice by manipulating sex chromosome constitution in culture.^[^ ^55^ ^]^ Y chromosome loss and X chromosome duplication facilitated the establishment of XX ESCs from XY ESCs, which were subsequently treated for in vitro oocyte generation.

From the initial development of PGCLCs to the generation of fully functional oocytes, each milestone represents a leap forward in our understanding of germline development and raises hope for broad applications. At the same time, challenges remain. To date, in vitro‐derived oocyte development does not match the efficiency of the ovary. For example, the success rate for obtaining healthy mice by 2‐cell embryo transfer ranges between 0.9% and 5.2% using in vitro‐derived oocytes, which is much lower than that of oocytes obtained from hormonally superovulated mice (61.7%).^[^ ^4^, ^5^, ^55^ ^]^ Efforts are ongoing to address genetic and epigenetic abnormalities observed in in vitro‐derived oocytes.^[^ ^56^ ^]^ The quest continues to elucidate the mechanism of low competence, refine culture steps, and enhance the efficacy to obtain embryos from oocytes derived from PSC cultures.

## CRITICAL DEVELOPMENTAL PROCESSES AND OPPORTUNITIES FOR IMPROVEMENTS

**FIGURE 4:**
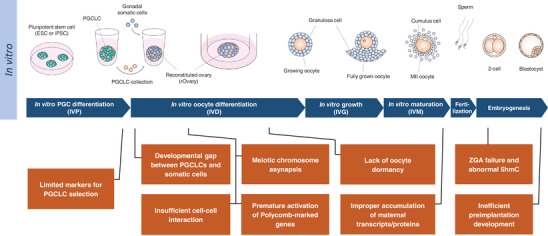
**Challenges in the in vitro culture system of oocyte generation**. (top) A schematic illustration of germ cell development from pluripotent stem cells (PSCs) in the culture system established by Hikabe et al.^[^ ^4^ ^]^ MII oocytes generated from PSCs can proceed to preimplantation development in vitro after fertilization with sperm. (bottom) The bottom part highlights key challenges (brown) in the culture system that affect the competence of oocytes for preimplantation development and embryogenesis. These challenges are listed along with the timeline of the in vitro oocyte development (blue). ESC, embryonic stem cell; iPSC, induced pluripotent stem cell; PGC, primordial germ cell; PGCLC, primordial germ cell‐like cell; ZGA, zygotic genome activation; 5hmC, 5‐hydroxymethylcytosine.

During oogenesis, oocytes undergo meiosis, and establish genetic and epigenetic information supporting their growth and further embryonic development. Oocytes also accumulate maternal factors that are required for fertilization and initiation of embryo development.^[^ ^34^, ^35^ ^]^ The quality of oocytes profoundly impacts the success of preimplantation development and subsequent embryonic viability. A robust protocol for generation of PSC‐derived oocytes has been reported, while only few researchers except from the original laboratories have, thus far, successfully recapitulated the oocyte generation.^[^ ^4^, ^56^ ^]^ This reflects the difficulty and skills required for the procedures and limited availability of suitable PSC lines for oocyte generation. Therefore, we next consider possible causes of low competence from our own work and the literature and discuss improvements (Figure 4).

*PGCLC development*: Recent studies, which reported successful in vitro generation of PSC‐derived mature oocytes, adopted the co‐culture of PGCLCs and gonadal somatic cells to form rOvaries.^[^ ^4^, ^55^, ^56^ ^]^ In these studies, PGCLCs were induced by the culture of EpiLCs for 6 days, termed d6 PGCLCs, while gonadal somatic cells were harvested from E12.5 fetuses. However, this co‐culture raises concerns regarding the developmental stage of PGCLCs, which is assessed by the levels of 5‐methylcytosine (5mC), the predominant form of DNA methylation. D6 PGCLCs attain an average 5mC level of 37%, mirroring the state observed in migrating PGCs at E9.5^[^ ^57^, ^58^ ^]^. The developmental gap between d6 PGCLCs akin to E9.5 PGCs and E12.5 somatic cells hereby arise in the rOvary, possibly leading to distortion in successive development. Therefore, the phase of PGC migration is currently not well recapitulated by the in vitro protocol. This concern is supported by our analysis, reporting abnormal premature activation of genes in growing oocytes during the IVD, possibly due to the premature removal of Polycomb repression.^[^ ^56^ ^]^ In fact, Yoshino et al. reported FOSLCs supported oogenesis only in a brief time window of FOSLC development.^[^ ^5^ ^]^ D6 PGCLCs developed to oocytes in a high number only when aggregated with FOSLCs that were differentiated to d5 or d6, and before the expression of the granulosa cell marker Foxl2. To close the developmental time gap between PGCLCs and gonadal somatic cells, extended culture of PGCLCs can be considered. Ohta et al. developed a culture method that allows further development and expansion of PGCLCs in the presence of agonists for cAMP signaling, rolipram, and forskolin.^[^ ^51^ ^]^ Importantly, extended culture of d4 PGCLCs for 7 days resulted in epigenetic reprogramming including the reduction of 5mC level to 6%, equivalent to that in E13.5 germ cells.^[^ ^57^, ^59^ ^]^ Compensation of developmental time could help to reconstruct the migration phase of PGCs and alleviate defects arising from premature development of PGCLCs during IVD.

*PGCLC selection*: Application of PSCs for producing a specific cell type often encounters a problem of aberrant differentiation or tumorigenesis.^[^ ^60^ ^]^ Oocyte generation is no exception. Selection and sorting of differentiated PGCLCs is a critical step to achieve oocyte development. Contamination with small amounts of undifferentiated cells can lead to abnormal outgrowth that interfere with rOvary development.^[^ ^56^ ^]^ Recent studies for successful gamete generation have combined two reporter transgenes, *Blimp1*‐mVenus (BV) and *Stella*‐ECFP (SC), for sorting PGCLCs.^[^ ^4^, ^5^, ^6^, ^49^, ^50^, ^55^, ^56^, ^61^, ^62^, ^63^ ^]^ For a more general application the use of PSCs without genetic modifications is appealing. Previous studies have identified the combination of two PGC surface markers, SSEA1 (stage‐specific embryonic antigen 1) and integrin‐β3, to sort BV positive PGCLCs, and successfully developed gametes from these PGCLCs.^[^ ^49^, ^50^ ^]^ However, we also observed the emergence of unexpected outgrowths using these surface markers.^[^ ^56^ ^]^ Subtle contamination by BV negative cells may have been present, potentially causing issues in subsequent rOvary development. Therefore, in addition to or as a substitute for SSEA1 and integrin‐β3, other PGC surface markers may contribute to more effective selection of PGCLCs. Mouse PGC surface markers, including integrins α3/α5/α6/αV/β1, E‐/P‐/N‐cadherins, PECAM‐1, Lewis X, c‐Kit and EPCAM, can be candidates for additional or substitute surface markers, some of which are also used for selection of human PGCLCs.^[^ ^50^, ^64^, ^65^ ^]^ Another consideration for improving PGCLC selection is the elimination of naive PSCs. Induction of germ cells is restricted to a narrow window of time during EpiLC differentiation similar to the window of germ cell competence in the early postimplantation epiblast.^[^ ^66^ ^]^ Recently, formative PSC cultures have been described that correspond to the early post‐implantation epiblast and maintain germ cell competence.^[^ ^67^ ^]^ Induction of PGCLCs from formative PSCs could help to eliminate undifferentiated cells. Although SSEA1 and integrin‐β3 positive PGCLCs have been obtained from formative PSCs, their potential for in vitro oogenesis remains to be examined experimentally in the future.

*Meiosis*: Meiosis is a critical process during which germ cells undergo homologous chromosome pairing, recombination, and a series of chromosome segregation, resulting in the formation of gametes. Meiosis in a mouse oocyte is completed at fertilization of the MII oocyte, which extrudes the second polar body and delivers a haploid maternal genome in the zygote. Defects in meiotic events have been observed in PSC‐derived oocytes in culture. Hikabe et al. reported that asynapsis between homologous chromosomes was observed in 53.8% of PSC‐derived germ cells at the pachytene stage, and 22.2% of PSC‐derived MII oocytes showed aneuploidy.^[^ ^4^ ^]^ This observation is reminiscent of aberrant expression of genes involved in cohesion and chromosome synapsis, including *Smc1b*, *Sycp3*, and *Hormad2*.^[^ ^68^, ^69^, ^70^ ^]^ Interestingly, such genes are part of an early meiotic gene program and transiently repressed by the Polycomb repressive complexes 1 and 2 (PRC1 and PRC2) following the loss of DNA methylation.^[^ ^71^, ^72^ ^]^ Polycomb‐mediated repression orchestrates the timely activation of such genes for entry into meiotic prophase. Intriguingly, Polycomb‐marked genes undergo premature activation in PSC‐derived oocytes during IVD,^[^ ^56^, ^73^ ^]^ suggesting that frequent asynapsis and aneuploidy in PSC‐derived oocytes may result from premature initiation of a meiotic program in in vitro grown oogonia during the IVD process. Extended culture of PGCLCs before the meiotic initiation in the rOvary, to further reduce the level of DNA methylation globally and of meiotic genes in particular, as described above, may overcome specific defects in meiosis reported before. The impact of in vitro germ cell development on endogenous repeat elements, their chromatin states, expression and possible detrimental functional roles, also awaits further characterization.^[^ ^71^, ^74^ ^]^

*Interaction between germ cells and somatic cells*: After migration, PGCs undergo intimate interaction with somatic cells in the gonad, leading to formation of cysts and subsequently follicles.^[^ ^21^, ^75^ ^]^ To mimic this development in vitro, co‐culture of PGCLCs with gonadal somatic cells or FOSLCs is implemented.^[^ ^76^ ^]^ This co‐culture system facilitates cell‐cell interactions within rOvaries between germ cells and mesoderm‐derived follicular somatic cells. Intra‐follicular communication between oocytes and surrounding somatic cells is crucial for the developmental competence of oocytes.^[^ ^26^ ^]^ Single oocyte transcriptomes revealed aberrant expression of genes involved in the production of extracellular matrix (ECM) and cell interaction in PSC‐derived growing oocytes^[^ ^56^ ^]^. Also, transcriptome analysis using transplanted gonads/ovaries suggested that the development of growing oocytes is heavily influenced by ECM, which also regulates oocyte dormancy.^[^ ^53^ ^]^ Considering that the formation of rOvaries requires cell dissociation and sorting of PGCLCs and somatic cells, essential components of the basement membrane and ECM might be lost. Observations of cells within the zona pellucida and the abnormally split structure of the zona might be indicative of improper cell‐cell distance or interaction.^[^ ^56^ ^]^ Studies have shown that development of a fragment of 14 days post‐partum (dpp) ovaries improved under a 3D culture supported by ECM‐rich Matrigel or hydrogels supplemented with ECM‐derived Arg‐Gly‐Asp (RGD) peptides.^[^ ^77^, ^78^ ^]^ Also, synthetic hydrogels with ECM‐sequestering fibers have been suggested to restore key cell‐cell interactions critical for oocyte growth.^[^ ^79^ ^]^ Targeting the restoration of extracellular components in rOvaries could enhance the culture system. Another potential approach to improving cell‐cell interaction is targeting intra‐follicular protein signals. Oocytes secrete GDF9 and BMP15, which are essential for intra‐follicular communication and follicle development.^[^ ^26^ ^]^ It is thought‐provoking that a transient supplement of GDF9 and BMP15 in the medium is necessary for PSC‐derived oocytes during IVG, whereas the culture of E12.5 gonads produced highly competent oocytes without this supplementation.^[^ ^80^ ^]^ A recent study demonstrated that amino acid substitution of GDF9, named Super‐GDF9, reduced its latency, promoting cumulus cell development and enhancing oocyte quality in vitro.^[^ ^81^ ^]^ Targeting the improvement of intra‐follicular protein signals, including application of Super‐GDF9, potentially promotes the competence of oocytes. A systematic pairwise analysis of transcriptomes of oocytes and associated follicular somatic cells of individual in vitro reconstituted versus naturally generated follicles may also provide insight into the variability in communication between germ and somatic cells among follicles and may suggest pathways affected under IVD and IVG conditions.

*Interventions to maintain or restore oocyte function*: Conceptually oocyte quality can also be improved by additional measures that either prevent defects from occurring or mitigate the consequences of defects. Our previous study has identified genes under epigenetic control were prematurely expressed in vitro.^[^ ^56^ ^]^ Pharmacologic manipulation of chromatin modifying activities could therefore be explored to prevent defects from arising. Chromatin remodeling during PGC development in vitro and in vivo has been studied including PRCs and DNA methyltransferases.^[^ ^71^, ^82^, ^83^ ^]^ It remains to be seen if temporal inhibition of these enzymes can correct for culture induced changes in epigenetic regulation. Also, we have characterized PSC‐derived oocytes and identified molecular changes and a frequent failure of ZGA as well as arrest at the 2‐cell stage.^[^ ^56^ ^]^ Identification of human infertility genes,^[^ ^84^, ^85^, ^86^ ^]^ understanding of phenotypes of mutations in germline genes in mice,^[^ ^87^, ^88^, ^89^, ^90^ ^]^ and progress in understanding the production of maternal factors for the oocyte cytoplasm^[^ ^35^, ^91^ ^]^ can provide ideas for mitigation strategies. For supporting genome activation and cell cycle progression methods for assisted oocyte activation have been considered in clinical settings.^[^ ^36^ ^]^ In addition to genetic mutations the metabolic state has been reported to affect the developmental potential of oocytes, maternal genome protection by *Stella* and genome activation.^[^ ^92^, ^93^ ^]^ In vitro‐derived oocytes have exhibited altered expression of mitochondrial genes,^[^ ^4^ ^]^ abnormal Stella localization, and cytoplasmic rather than nuclear localization of pyruvate dehydrogenase.^[^ ^56^ ^]^ Therefore, metabolic support by activation of pyruvate dehydrogenase, which is required for providing co‐factors for histone acetylation, might be worth exploring to mitigate the observed defects. Lastly, activation of genomic retrotransposon derived repeat elements has been shown to cause DNA damage and contribute to the loss of a large fraction of primordial follicles from the ovarian reserve in mice.^[^ ^94^ ^]^ The observation that mitigation of DNA damage using reverse transcriptase and CHK2 kinase inhibitors led to an increase in the number of follicles suggests a potential intervention for improving oocyte yield and quality.

*Application of donor oocytes with replacement of their nuclei*: Cytoplasmic components of oocytes are crucial for competence of fertilization and subsequent embryogenesis. However, RNA‐seq analyses have indicated abnormal transcript accumulation in in vitro‐derived oocytes, caused by aberrant expression throughout the oocyte development during IVD and IVG.^[^ ^4^, ^56^ ^]^ An approach to address this issue is utilizing healthy ooplasm via somatic cell nuclear transfer (SCNT), a technique pioneered in the 1990s.^[^ ^95^, ^96^, ^97^ ^]^ Recent studies have applied SCNT to reconstruct functional mouse oocytes by replacing donor oocyte nuclei. A notable study has recently reported the haploidization of the somatic cell genome in oocytes and its application in constructing functional oocytes.^[^ ^98^, ^99^ ^]^ Replacing meiotic spindles in donor MII oocytes with fibroblast or cumulus cell nuclei in the G0/G1 phase led to the segregation of somatic homologous chromosomes in some embryos after fertilization with sperm. Remarkably, the production of live offspring has been reported from these somatic‐sperm embryos at rates ranging from 0% (0/118) to 3.7% (3/81) of transferred embryos, depending on the combination of mouse strains used.^[^ ^98^ ^]^ Although mechanisms remain unclear, somatic cell haploidy offers a potential alternative for generating PSC‐derived oocytes. Another innovative approach employs haploid ESCs as substitutes for the oocyte genome.^[^ ^100^, ^101^ ^]^ Haploid ESCs are stem cell lines derived from the inner cell mass of parthenogenetic or androgenetic haploid blastocysts, possessing a single set of chromosomes.^[^ ^102^, ^103^, ^104^, ^105^ ^]^ It has been demonstrated that parthenogenetic haploid ESCs can reconstruct embryos in place of the maternal genome due to their haploidy, resulting in the production of fertile mice in 0.7% (2/290) of transferred embryos.^[^ ^100^ ^]^ Additionally, haploid ESCs can serve as a substitute for the paternal genome.^[^ ^104^, ^105^, ^106^, ^107^ ^]^ Deleting targeted differentially methylated regions (DMRs), such as *H19*‐, *IG*‐, and *Rasgrf1*‐DMRs, facilitated paternal expression of imprinted genes.^[^ ^101^, ^108^ ^]^ Both parthenogenetic and androgenetic haploid ESCs, with or without these deletions, have demonstrated potential as sperm substitutes following intracytoplasmic injection into oocytes.^[^ ^104^, ^105^, ^106^, ^107^, ^109^ ^]^ Using them as sperm substitutes has resulted in live offspring at higher rates, with up to 20.2% (402/1993) of transferred embryos.^[^ ^109^ ^]^ However, these approaches to replace gametic genome still face several limitations. One significant limitation is the requirement for donor oocytes, which differs markedly from the generation of oocytes from PSCs in culture. Also, the successful construction of functional oocytes through the haploidization of the somatic cell genome highly depends on the proper segregation of homologous chromosomes.^[^ ^98^ ^]^ Sequence homology between homologous chromosomes is critical for segregating the haploid genome of somatic cells, suggesting the necessity of somatic cells from inbred strains, which limits applicability to certain species and excludes humans.^[^ ^99^ ^]^ Another limitation is genomic imprinting when applying somatic cells or haploid ESCs as gametic genome substitutes. Genetic modification of DMRs in haploid ESCs is sometimes necessary to adjust genomic imprinting for effectively replacing the oocyte or sperm genome.^[^ ^101^, ^106^, ^107^ ^]^ Lastly, unexpected polyploidy has been observed in embryos constructed using haploid ESCs as sperm substitutes, likely caused by segregation defects of M‐phase chromosomes or diploidization of the haploid genome.^[^ ^110^ ^]^ Despite these challenges, the approach to reconstruct oocytes or embryos through nuclear replacement offers considerable advantages depending on the context. The cytoplasm of oocytes significantly influences competence of embryogenesis, and using donor oocytes allows to compare the quality of cytoplasmic components to that of PSC‐derived oocytes. Also, haploid ESC genomes can be directly modified in culture due to their self‐renewal capacity, making them a promising tool for genetic investigations, particularly in biological studies such as genetic screening.^[^ ^109^, ^111^, ^112^ ^]^

## CONCLUSIONS

Obtaining fully functional oocytes through in vitro culture opens new avenues in research and opportunities for medicine, infertility treatment, and animal conservation.^[^ ^7^ ^]^ Reconstitution of developmental processes has been key to making functional gametes and illustrates that pathways of animal embryogenesis can be recreated in culture. These achievements provide an unprecedented test of our knowledge and new experimental opportunities raising hopes that similar approaches can be extended to a wide range of lineages. However, predictions are difficult as embryogenesis is a complex process. Reconstruction of glomeruli of the kidney^[^ ^3^ ^]^ and differentiation of hematopoietic stem cells from pluripotent stem cells are impressive examples.^[^ ^8^ ^]^ However, for the latter engraftment and support of life‐long hematopoiesis remain to be demonstrated. Oogenesis offers unique advantages that might not apply to other lineages. Firstly, oocytes are large cells and can be easily identified and isolated with a glass pipette. Secondly, epigenetic reprogramming is integral to germline and early embryo development. Therefore, epigenetic defects might be corrected after fertilization when in preimplantation development most epigenetic marks are removed from the gametic genomes.^[^ ^30^ ^]^ Indeed, SCNT experiments illustrate that the oocyte has the ability to reprogram a wide range of somatic cell nuclei resulting in the production of cloned mice.^[^ ^96^ ^]^ These unique features might provide advantages to extend in vitro gametogenesis to other species including humans. Nevertheless, the application of in vitro gametogenesis to other species remains challenging since the mechanisms underlying germ cell development differ between species.^[^ ^113^ ^]^ For example, signaling and transcriptional networks for PGC specification differ between mice and humans.^[^ ^7^ ^]^ While PGCLCs and oogonia have been successfully induced from PSCs in human by in vitro culture, the generation of oocytes has not been reported at present.^[^ ^114^, ^115^, ^116^, ^117^ ^]^ Also, if PSC‐derived human oocytes are generated in the future, their application will require extensive and careful consideration. Similar to human embryo models formed from stem cells, ethical considerations are paramount.^[^ ^118^ ^]^ A strategy to assess oocyte quality and the development of an ethical framework are necessary for clinical applications.^[^ ^113^, ^118^ ^]^ Nevertheless, benefits of in vitro gametogenesis across species are immense for the broader community. Recent advances in cell culture technology have the potential to open new vistas and establish a new paradigm.

## CONFLICT OF INTEREST STATEMENT

The authors declare to have no conflict of interest in the context of this article.

## Data Availability

Data sharing is not applicable to this article as no new data were created or analyzed in this study.
